# Supplementary material for: Body Size and Behavioural Plasticity Interact to Influence the Performance of Free-Foraging Bumble Bee Colonies
Source: Insects. 2021 Mar 10;12(3):236. doi: 10.3390/insects12030236 (PMC8001989; doi:10.3390/insects12030236)
Supplement: Supplementary file 1 [file insects-12-00236-s001.pdf]

Body size and behavioural plasticity interact to influence the performance of free-foraging bumble bee colonies

Supplementary Material

Jacob G. Holland [1,*,†], Shinnosuke Nakayama [2], Maurizio Porfiri [3,4,5], Oded Nov [6] and Guy Bloch [1,7,*]

[1] Department of Ecology, Evolution & Behaviour, Alexander Silberman Institute of Life Sciences, Hebrew University of Jerusalem, Jerusalem 91904, Israel
[2] Department of Mechanical and Aerospace Engineering, New York University Tandon School of Engineering, Brooklyn, NY 11201, USA; shinn407@gmail.com
[3] Center for Urban Science and Progress, New York University Tandon School of Engineering, Brooklyn, NY 11201, USA; mporfiri@nyu.edu
[4] Department of Mechanical and Aerospace Engineering, New York University Tandon School of Engineering, Brooklyn, NY 11201, USA
[5] Department of Biomedical Engineering, New York University Tandon School of Engineering, Brooklyn, NY 11201, USA
[6] Department of Technology Management and Innovation, New York University Tandon School of Engineering, Brooklyn, NY 11201, USA; onov@nyu.edu
[7] The Federmann Center for the Study of Rationality, The Hebrew University of Jerusalem, Rehovot 7610001, Israel
* Correspondence: jacob.holland@ed.ac.uk (J.G.H.); guy.bloch@mail.huji.ac.il (G.B.)
† Current address: Institute of Evolutionary Biology, University of Edinburgh, Edinburgh EH9 3FL, UK

SUPPLEMENTARY METHODS

Further detail for colony foundation and male production

In Trial 1, colonies were founded by unrelated queens that did not undergo diapause. These queens underwent two CO_2 narcosis treatments which circumvent diapause [1,2] and were provided with a conspecific ‘stimulatory’ worker in order to stimulate them to found colonies in the rearing facility. During this trial, several colonies produced males on or before day 25 of the experiment (n = 37 males across 7 colonies), meaning that they were produced from eggs laid before treatment began, given that the normal egg to adult developmental time is around 26 days, e.g. [3,4]. Early male emergence is common in industry-produced colonies in which the queens do not experience natural diapause [5]. It is also possible that the introduced stimulatory workers laid eggs. We excluded the possibility that these males were caused by inbreeding, which can result in diploid males occurring [6], because the ratio of males to workers in the first 25 days was significantly different from 1:1, as expected for colonies with diploid males (goodness of fit tests, all p-values < 0.01 across colonies; one colony produced ~80% males within this time period and the others produced 10% or less). Note that, as mentioned in the Methods (*Colony Maintenance and Treatment*), these early produced males were replaced with workers, which should have mitigated any detrimental effects of male production (i.e. males acting as a burden on colony resources). The colony which produced a high proportion of early males eventually shifted to a more typical production schedule with mostly females (similar to the other colonies).

In the second trial, we undertook several measures to reduce genetic variation among worker bees and to reduce early male production. Specifically, we used colonies that were all headed by sister-queens (i.e., emerging from a single mother colony) which were mated by brother-males (from another unrelated colony). This should mean that workers across different colonies had an average relatedness value of 0.625, which is almost as high as workers within the same colony (r = 0.75). We reasoned that this might help to control for variation between

colonies in performance, aside from the effects of treatment. The queens also underwent a short (3-week) diapause (which more closely simulates natural conditions) before CO_2 treatment, and the conspecific stimulatory workers were removed or replaced (as necessary) after 1 week. These procedures substantially reduced the emergence of early males in this trial (n = 1 male from a single colony).

In Trial 1, the mean eclosion date of the first worker (before receipt of colonies from supplier) was estimated to be four days before receipt of the colonies, i.e. 10th May 2015 (given that the number of workers upon receipt of the colonies was similar to Trial 2, where the mean first workers eclosion was known to have occurred four days before receipt). In Trial 2, the mean first worker eclosion date was known to be 22nd June 2016. Thus, four days should be added to the reported experimental days to calculate the date from first worker eclosion (in both trials).

The differences between trials are summarised in Table S1.

Table S1. Summary of differences between the trials.

	Trial 1	Trial 2
**No. Colonies**	9	11
**Colonies closely related**	No	Yes
**Colony foundresses experienced diapause**	No	Yes
**Dates**	May-July 2015	June-August 2016
**Dead workers replaced periodically**	Only during the initial part of experiment	No
**Continued provision of resources**	Until Day 22 (pollen) / Day 24 (syrup)	Throughout experiment

Further detail of treatments and colony maintenance

Starting with the initial workers, every 1-2 days throughout the experiment, all previously unmeasured workers from all focal colonies were collected, measured and classed as small, medium or large. Most of these workers were then marked and redistributed among the focal colonies in accordance with colony treatment, along with a smaller number of workers from non-focal donor colonies (Trial 1, 9%; Trial 2, 19% of all introduced workers were from genetically unrelated donor colonies). The mean size of introduced workers on any given day was also kept approximately the same between homogeneous and diverse colonies (mean ± SD daily difference between mean marginal cell lengths of workers introduced to the two treatments; Trial 1 = 0.063 ± 0.06 mm; Trial 2 = 0.021 ± 0.024 mm).

Until the time of colony connection to the outside, any males or dead workers found in colonies were replaced by similarly sized workers as part of worker redistribution the following day, in order to mitigate any effect of male production or mortality at this stage on colony performance. Colonies were initially provided with ad libitum inverted sugar syrup and pollen (mixed to a

thick paste with a small amount of syrup), provided by the colony supplier. After colonies were connected to the outside, 0.5-1 g of pollen and/or 1-5 ml of syrup was provided directly into food cells of each colony every 1-5 days, depending on stored food levels, with equal amounts always supplied to all colonies on any given day. In Trial 1, provisioning with pollen and syrup was stopped at Day 22 and 24 respectively (i.e. 10 and 12 days after the first colonies were connected). In Trial 2, which was conducted later into the summer, a small amount of pollen and syrup continued to be provided, equally across colonies, throughout the duration of the experiment. The provision of this supplementary food was continued in Trial 2 to prevent the colonies from collapsing, since almost all colonies were struggling to grow (i.e. only a slight or no increase in total workers beginning shortly after the colonies were connected). The amount provided was not sufficient to support the colonies completely (indeed all colonies remained fairly weak) and, for most colonies across both treatments, was only a small proportion of the total food they collected. Thus, all colonies needed to forage for food, and it is unlikely that this supplementary food in Trial 2 would compensate for any effect of treatment on colony performance.

Further detail on behavioural classification

Detailed descriptions of in-nest tasks are as follows: **tending brood* = sustained inspection of brood cells with mandibles and antennae, or feeding of larvae (type of brood tended, larva or pupa, was also recorded in Trial 2); **constructing* = the manipulation by mandibles of wax substrate, either by building/ modifying food cells, or by forming connections between food/brood cells, or otherwise the manipulation of other nest substrate (pulp from cardboard base of nest – recorded separately in Trial 2); *grooming* = sustained self-grooming of legs, thorax, head or proboscis; **fanning* = sustained (presumed thermoregulatory) fanning of wings, whilst standing still and raised on legs, usually standing on brood comb or wall of nestbox (and not directed towards other individuals); *feeding* = feeding self with proboscis in food cell; **depositing food* = obviously depositing nectar or pollen into food cell; *egg-tending* = sustained inspection of egg cells, eating eggs or laying eggs (-each recorded separately);

aggression = antagonistic behaviour towards other workers or the queen (buzzing, antennating, darting, mandibulating/ biting or stinging attempts –each recorded separately); *walking* = actively moving, but with no obvious or sustained other behaviour, often characterised by moving swiftly over the nest comb or around the periphery of nest, and only stopping briefly (<2 seconds) to inspect food or brood cells; *inactive* = stood still with no clear other behaviour, often characterised with head in down position but with mandibles or antennae not moving, commonly consistent with sleep (but at other times apparently alert) [7]. In Trial 2 only, **incubating brood* was also distinguished, characterised by standing with body tightly pressed against brood and with abdomen pulsating.

Data cleaning

Occasionally, errors or uncertainties in tag identification stemmed from difficulty seeing the exact tag ID, or from human error (e.g. recording the wrong color or number). This represented 8.8% and 5.1% of the total records in Trials 1 and 2, respectively. In these cases it was important to assign the tags to the most likely candidate tag ID where possible, using strict criteria (see below), in order to ensure the data were not biased against certain tags or certain behaviours. If no credible alternative tag identity was clear, or if two or more tags were equally credible, the tag record could not be reinterpreted and was discarded from the dataset. This was the case in 2.8% in both Trials 1 and 2. Candidate tag credibility was determined by several parameters: the candidate tag ID must have been introduced into the focal colony, OR must have been an established drifter to the focal colony. In addition, the tag must have either been noted as a possible alternative at the time of recording, OR must have been a color-based mistake (i.e. with the same number), OR must have been similar in each of its digits (e.g. a 3 is mistakable for an 8). Any tag identities which were spotted in the same in-nest scan as the focal tag were assumed not to be a credible alternative (since a tag should only have been recorded once per in-nest scan). A tag identity which was recorded in other in-nest scans during the same session or day was taken as evidence in support of the tag being credible, because it showed that that bee was present at around the same time as the focal bee. The

same is true for tag identities recorded during a single foraging scan (since multiple records of a forager were permitted during foraging scans, and foraging workers often foraged in back-to-back foraging bouts).

Further detail in statistical modelling

For LMMs and GLMMs, the ‘lmer’ or ‘glmer’ functions from R package ‘lme4’ (Bates et al. 2015), or the ‘glmmTMB’ function from the R package ‘glmmTMB’ were used, with model checking using the ‘Dharma’ package. In all within-trial linear models, non-significant fixed effects were removed from the model in reverse significance order (interaction terms before subsumed main effects), with p-values determined by likelihood ratio tests. For any fixed effects remaining in the minimally adequate model (i.e. where all terms significant or subsumed within significant interaction terms), reported *p*-values are generated from ANOVA using degrees of freedom derived from Satterthwaite’s method. In the models for comparing the number of food cells, GLMMs were used with a Poisson error distribution with a log link function, which is usually appropriate for count data as a response variable [8]. Day was included as both fixed and random predictor variables, to allow models to include a general effect of time (fixed effect) as well as day-to-day variation (random effect). The equivalent models without the random intercepts for Day had either higher or equivocal AIC values, and so Day was included for all four GLMMs. For the fixed effect, Day was mean-centred (i.e. the mean Day value of all observations, 44.6, was subtracted from each Day value; e.g. Day 50 becomes Day 5.4 for the purposes of the model). This allows for meaningful interpretation of main effect of treatment in the presence of a Treatment x Day interaction term, because the effect of treatment was compared at the mean day of observations, rather than at default intercept at Day 0 (which would be meaningless because colonies were not foraging at Day 0 of the experiment). For the pollen models, zero-inflated GLMMs were used (with the glmmTMB package) to account for the disproportionately large number of zeros in the pollen cell count data. For the ZI part of models, AIC value was used to find the best of: all terms from the conditional model, only fixed terms from the conditional model (which was the best for the Trial

1 model), or interaction term only (which was the best for the Trial 2 model). In all models, the significance of fixed factors refers to the difference in intercept between the two factor levels (e.g. homogeneous vs. diverse treatment). The significance of the slope of fixed covariates (e.g. 'Day') refers to the slope for the diverse treatment. The significance of a covariate x treatment interaction refers to the slope of the homogeneous treatment, as compared to the diverse treatment. Intraclass correlation coefficients (ICC), which give an indication of the variation in the response which can be explained by variation in the random effects were calculated using the "sjstats" package [9]. For the (stored food) models with crossed random effects, adjusted ICCs were used which take into account the variance of all random effects.

For the cross-trial models, four separate linear model sets were employed, one for each response variable. For each model set, the following terms were selected for the full model: trial, treatment, mean inactivity, total foraging rate, total foraging rate x treatment. In order to allow treatment to be interpretable given the interaction with foraging rate, the foraging rate was mean-centred (using mean value of 0.49 observations per minute). Due to the limited sample size (number of colonies), reduction in the number of terms was considered necessary to produce useful models. Model selection was performed by comparing the AICc (Akaike's Information Criterion corrected for small sample size) values of the full set of models with all combinations of these terms, using the R function 'dredge' (MuMIn package; Barton 2009). The only stipulation was that the model must include the Trial term, given the differences in results between the two years. In all cases, the delta AICc value of the selected model was less than 4 (range = 0.2 - 3.2; i.e. versus the best model when the trial term was allowed to be excluded), representing a reasonably well supported model (Burnham et al. 2010). Note that trial was modelled as a fixed factor rather than random factor, since there were only two levels.

SUPPLEMENTARY RESULTS

Pollen and nectar cells over time

The results of the number of full nectar and pollen cells over time (Figure S1) are fully described in the main text.

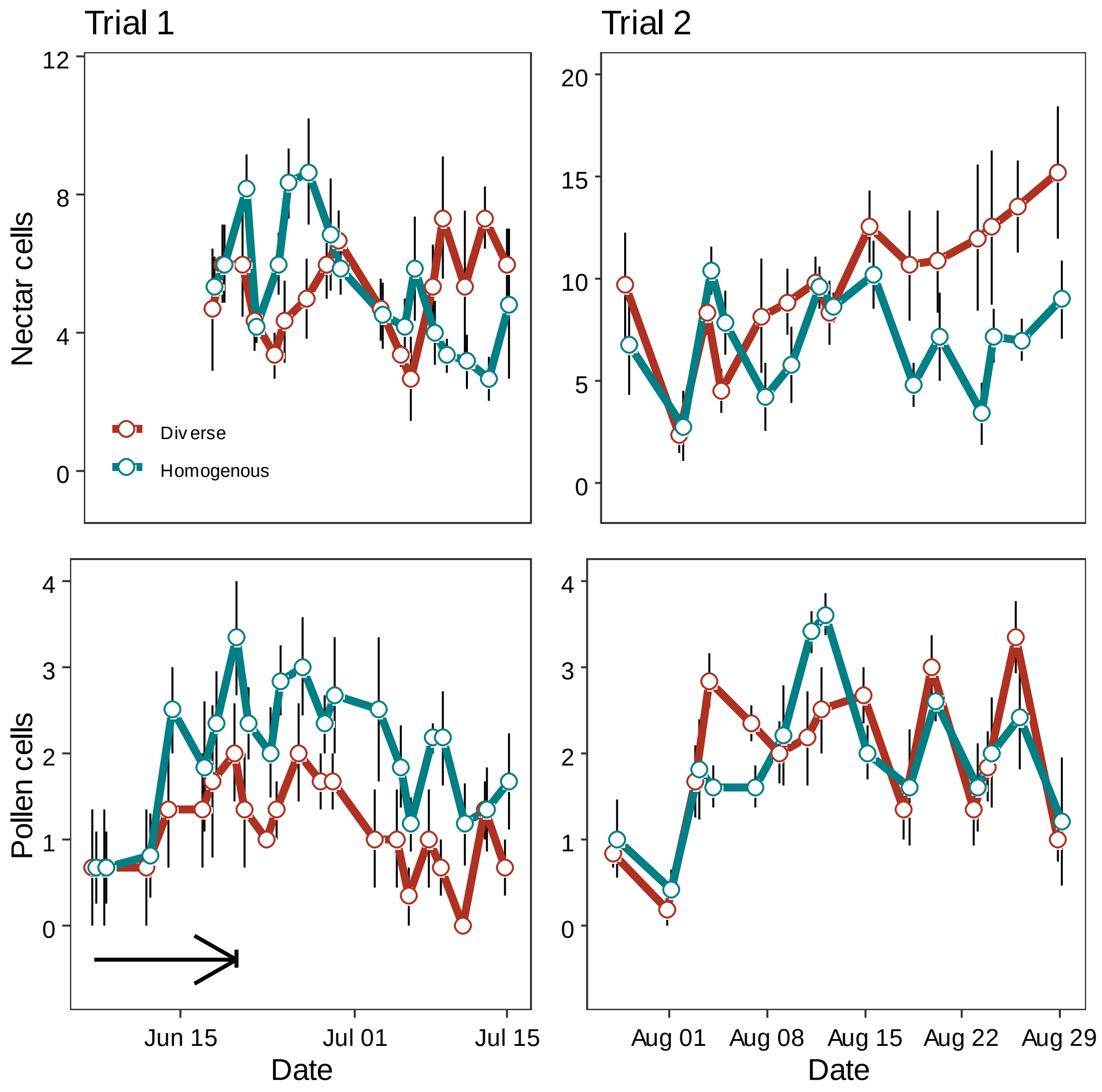

Figure S1. Estimated number of full pollen and nectar cells over time in size-diverse and -homogeneous colonies. Shown are mean ± SE for Trial 1 (left column) and Trial 2 (right column). The numbers were estimated based on visual inspection of cells once every 1-3 evenings. Colonies were connected to the outside environment on 25 or 26 May 2015 (Trial 1) or on 12 July 2016 (Trial 2). In top left plot, the blank area before 17 June indicates period when some supplemental syrup remained in colonies and could not easily be distinguished from collected nectar. In the lower left plot, the thick black arrow indicates a period in Trial 1 when workers were introduced depending on mortality levels in an attempt

to keep colony size similar between treatments at this stage (there was no corresponding period in Trial 2).

Pollen and nectar foraging rates

Further to comparing the rate of leaving the nest to forage and returning to the nest (see Results), we also compared the rate of bees returning to the nest with or without (assumed to be mostly nectar foraging) pollen. For both measures, there was no statistically significant difference between treatments (Mann Whitney U tests; without pollen: Trial 1, U = 2.5, n = 8, p = 0.15; Trial 2, U = 16, n = 11, p = 0.93; with pollen: Trial 1, U = 3, n = 8, p = 0.2; Trial 2, U = 22, n = 11, p = 0.23; Figure S2).

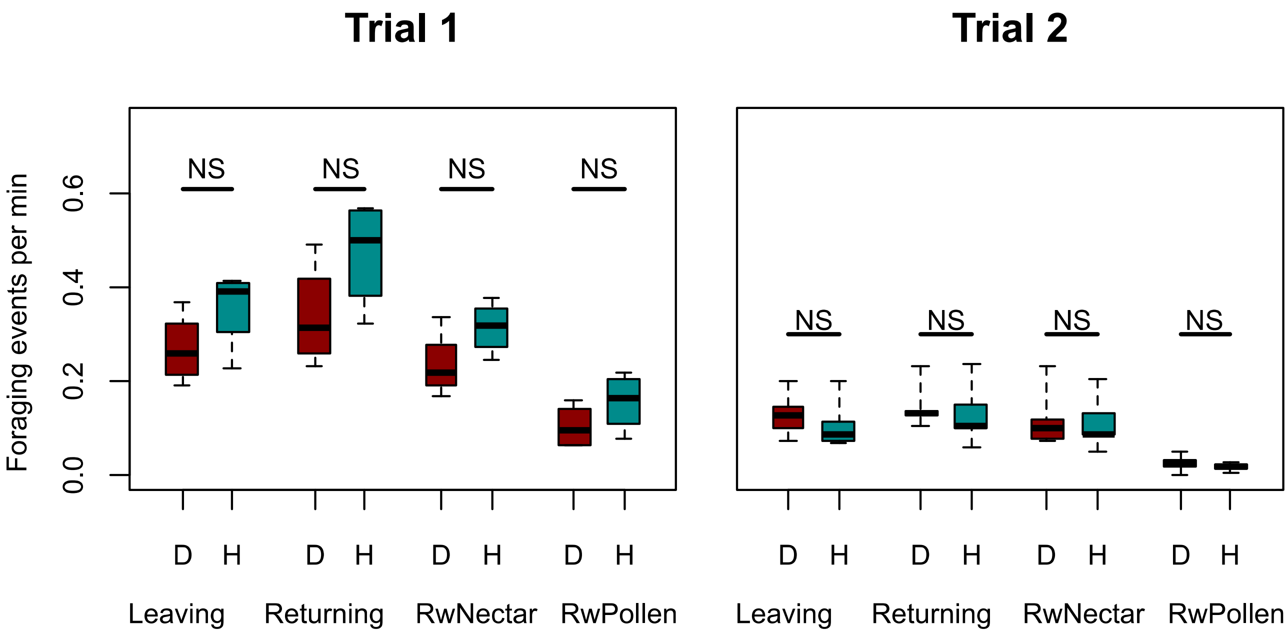

Figure S2. The total foraging records in size-diverse and -homogeneous colonies. Colony totals shown separately for homogeneous ('H') and diverse treatments ('D'). Foraging records separated into observations in which an individual was: 'Leaving' = leaving the nest by darting out, assumed to be on route to forage; 'Returning' = returning to the nest (total); 'RwNectar' = returning to the nest without pollen, so assumed to be nectar foraging; 'RwPollen' = returning to the nest with pollen attached to

pollen baskets, demonstrably pollen foraging. Thick black lines = medians; boxes = interquartile ranges; dashed whiskers = ranges. NS = p > 0.05, Mann Whitney U Test.

The sizes of workers produced by each treatment

The effect of each treatment on the mean body size of workers emerging in each colony is fully described in the main text and summarised in Figure S3.

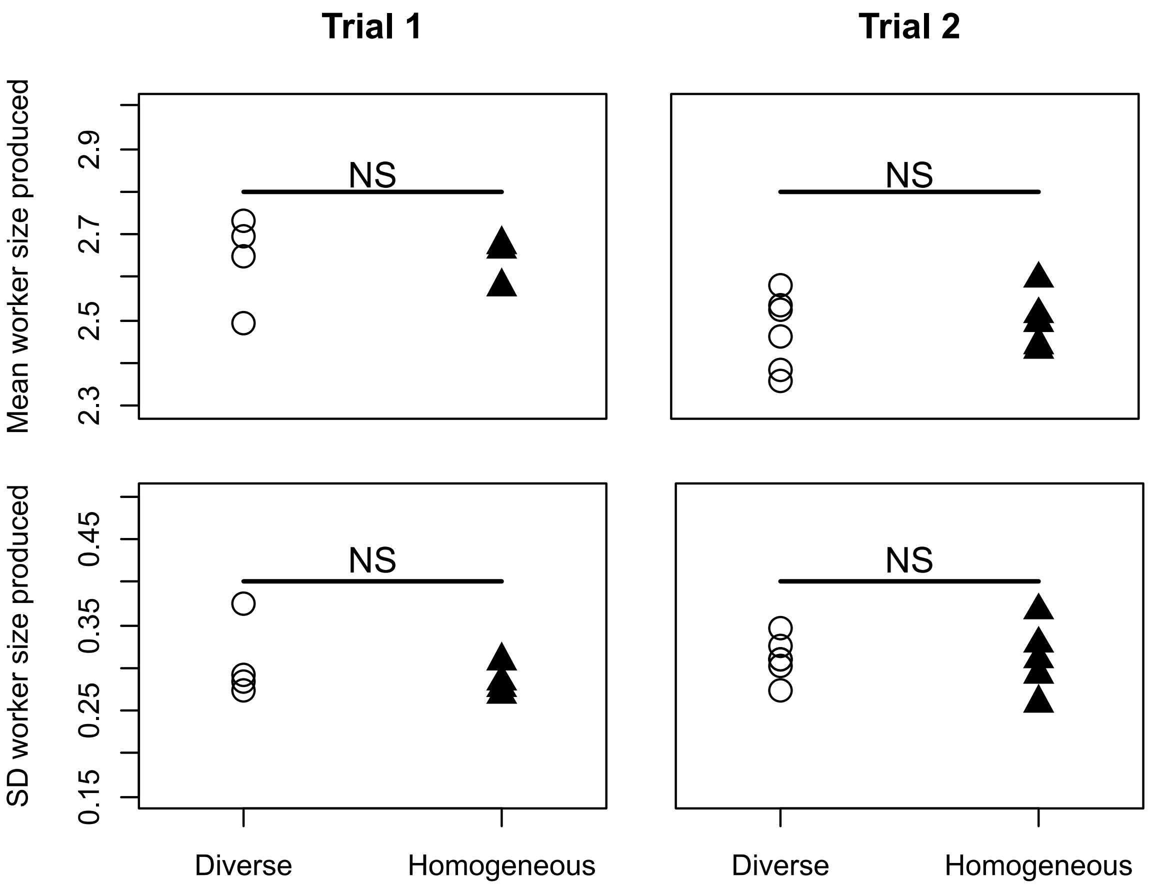

Figure S3. The influence of colony body size distribution on the body size of newly emerging bees. Upper row plots show the mean size of worker produced over the course of the experiment per colony, for each treatment. Lower row plots show the standard deviation of worker size produced over the course of the experiment per colony, for each treatment. NS = p > 0.05, Mann Whitney U Test.

The correlation between nursing and foraging activity of individual bees

When analysing each treatment separately, a significant negative correlation between the frequency of nursing and foraging observations per worker was found for the two treatments in Trial 1 (Pearson's correlation; diverse treatment, r = -0.25, n = 119, p = 0.006; homogeneous treatment, r = -0.25, n = 132, p = 0.004), while for Trial 2 the relationship fell short of significance (diverse treatment, r = -0.11, n = 273, p = 0.068, homogeneous treatment, r = -0.10, n = 282, p = 0.103).

The body sizes of nurses and foragers across treatments

In both trials, workers classed as 'foragers' were significantly larger in the diverse treatment than in the homogeneous treatment (Mann Whitney U tests; Trial 1, U = 421, n = 47, p = 0.002; Trial 2, U = 1740, n = 92, $p < 1 \times 10^{-8}$; Figure 5). Workers classed as 'nurses' were smaller in the diverse treatment than in the homogeneous treatment in Trial 1 (Mann Whitney U test; U = 437, n = 71, p = 0.026), but not in Trial 2 (Mann Whitney U test; U = 3710, n = 167, p = 0.46). In both trials, body size of bees classed as 'intermediate' was similar for bees in the diverse and homogenous treatment colonies (Mann Whitney U tests; Trial 1, U = 2121, n = 133, p = 0.70; Trial 2, U = 10929, n = 299, p = 0.74).

References for supplementary material

1. Roseler, P.F. A technique for year-round rearing of *Bombus terrestris* (apidae, bombini) colonies in captivity. *Apidologie* **1985**, *16*, 165-169.
2. Tasei, J.N. Effect of different narcosis procedures on initiating ovipostion of prediapausing *Bombus terrestris* queens. *Entomologia Experimentalis Et Applicata* **1994**, *72*, 273-279.
3. Bloch, G. Regulation of queen-worker conflict in bumble-bee (*Bombus terrestris*) colonies. *Proceedings Of The Royal Society Of London Series B-Biological Sciences* **1999**, *266*, 2465-2469.
4. Duchateau, M.J.; Velthuis, H.H.W. Development and reproductive strategies in *Bombus* colonies. *Behaviour* **1988**, *107*, 186-207.
5. Velthuis, H. The historical background of the domestication of the bumble-bee, *Bombus terrestris*, and its intorduction into agriculture. In *Pollinating bees - the conservation link between agriculture and nature*, Kevan, P.; Imperatriz Fonseca, V.L., Eds. Ministry of the Environment: Brasilia, **2002**.
6. Duchateau, M.J.; Hoshiba, H.; Velthuis, H.H.W. Diploid males in the bumble-bee *Bombus terrestris* sex determination, sex alleles and viability. *Entomologia Experimentalis Et Applicata* **1994**, *71*, 263-269.
7. Nagari, M.; Gera, A.; Jonsson, S.; Bloch, G. Bumble bee workers give up sleep to care for offspring that are not their own. *Current Biology* **2019**, *29*, 3488-3493.
8. Crawley, M.J. *The r book*. John Wiley & Sons Ltd: Chichester, England, **2007**.
9. Lüdecke, D. _sjstats: Statistical functions for regression models (version 0.18.1), 2021.
